# Optimization of Influential Variables in the Development of Buprenorphine and Bupivacaine Loaded Invasome for Dermal Delivery

**DOI:** 10.34172/apb.2021.060

**Published:** 2020-09-19

**Authors:** Soraya Babaie, Arezou Taghvimi, Mohammad Charkhpour, Amir Zarebkohan, Peyman Keyhanvar, Hamed Hamishehkar

**Affiliations:** ^1^Pharmaceutical Analysis Research Center, Student Research Committee, and Faculty of Advanced Medical Science, Tabriz University of Medical Sciences, Tabriz, Iran.; ^2^Biotechnology Research Center, Tabriz University of Medical Sciences, Tabriz, Iran.; ^3^Department of Pharmacology and Toxicology, Faculty of Pharmacy, Tabriz University of Medical, SciencesTabriz, Iran.; ^4^Department of Medical Nanotechnology, Faculty of Advanced Medical Science, Tabriz University of Medical Science, Tabriz, Iran.; ^5^Research Center for Pharmaceutical Nanotechnology, Stem Cell and Regenerative Medicine Institute, and Department of Medical Nanotechnology, Faculty of Advanced Medical Science, Tabriz University of Medical Science, Tabriz, Iran.; ^6^Drug Applied Research Center, Tabriz University of Medical Sciences, Tabriz, Iran.

**Keywords:** Buprenorphine hydrochloride, Bupivacaine hydrochloride, Invasome, Analgesic, Transdermal

## Abstract

***Purpose:*** Hydrophilic drugs are extensively applied in clinical applications. Inadequate dermal penetration of these drugs is a great challenge. Incorporation of drugs into nano-carrier systems overcomes lower penetration drawbacks. Invasomes are novel nano-carrier systems which enhance transdermal penetration by using terpene and ethanol in their structures. buprenorphine and bupivacaine hydrochlorides are two potent analgesic drugs that are loaded simultaneously in the nano-invasome structure as opioid and non-opioid drugs.

***Methods:*** The full factorial experimental design was used for planning and estimating optimum formulations of invasome systems. Three influential factors like terpene type, terpene concentration and preparation method were comprehensively analyzed for achieving high encapsulation efficiency (EE) and optimum size.

***Results:*** The mean sizes of designed invasomes were in the range of 0.39-5.86 µm and high values of EE and loading capacity (LC) were reported as 98.77 and 19.75 for buprenorphine-loaded invasome, respectively. Zeta potential measurements confirmed that the obtained high value of EE might be as a result of reversible ionic interactions between positively charged drugs and negatively charged phospholipidic part of invasome structure. Another characterization of the prepared formulations was carried out by Fourier transform infrared (FTIR), X-ray diffraction (XRD) and dynamic light scattering (DLS) technique.

***Conclusion:*** The satisfactory obtained results of formulations encourage researchers to get optimum topical analgesic formulations with potent and rapid onset time properties required in invasive cutaneous procedures.

## Introduction

**Figure 1:**
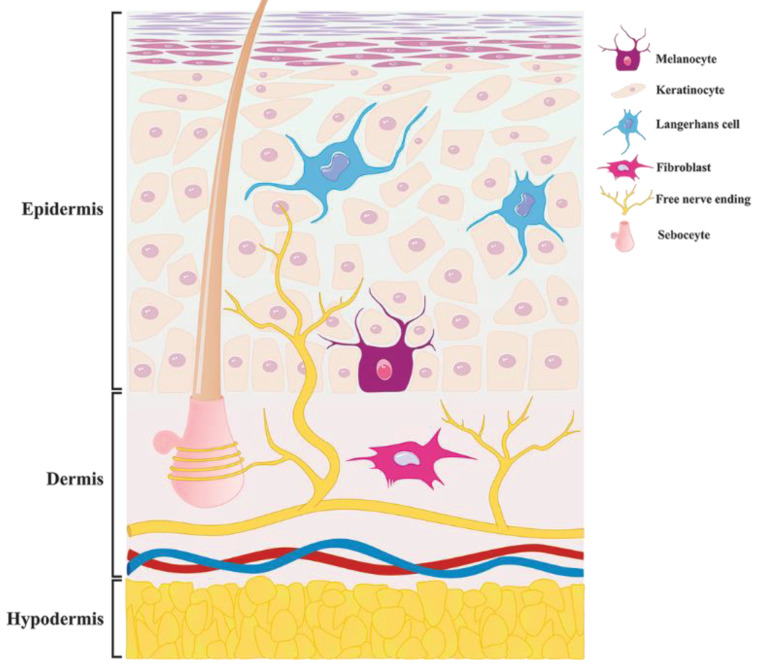
Distribution ofall opioid receptors in the skin cells i.e. melanocyte, keratinocyte, Langerhans cell, fibroblast, sebocytes and Free nerve ending.

The skin surface is extremely sensitive because of comprehensive distribution of pain receptors.^1^ When the injury happens, pain signals are produced by activation of inflammatory responses and the release of vasoactive neuropeptides. These events are generated by nerve endings stimulation concentrated on the surface of skin.^2^ Different types of invasive procedures like venipuncture and skin biopsy are painful operations.^3^ The pain sensed in these operations is unpleasant especially in children. Therefore, different anesthetic options are introduced by physicians.^4^ Anesthetic infiltration is a conventional intradermal anesthetic method. Needle panic and distorted injection site are some drawbacks of this method.^5^ To overcome the mentioned obstacles, designing novel drug delivery systems and effective pain relief drugs are important attempts in blocking pain at peripheral sites. Organizing topical analgesic formulations affect the peripheral nerves and diminish the pain perception.^6^ Opioids and non-opioid drugs such as local anesthetics are two groups of analgesics that are commonly used for dermal analgesia. Five classes of opioid receptors have been identified in the skin and clinical studies which have demonstrated the cutaneous analgesic effect.^7-9^ Skin opioid receptors have been discovered in keratinocytes,^10^ melanocytes, sebocytes,fibroblasts, immune cells and skin free nerve ending (Figure 1).^11^ Among present opioids, buprenorphine is highly applied as an effective and well-tolerated opioid.^12^ Local anesthetics generate anesthesia by blocking sodium ion channels and stops neu­ral conduction signals in neural membranes.^13^ Eutectic mixture of local anesthetics (EMLA^®^) consists of prilocaine and lidocaine that is the first commercial local anesthetics product.^14^ Bupivacaine is categorized as an amidic local anesthetic which is extensively applied in chronic and acute pain.^15^ Application of opioid and non-opioid anesthetics concurrently may provide synergistic effect and also may block simultaneously opioid receptors and nerve endings which are concentrated on the skin. Thereby, rapid onset time and potent analgesic effects are obtained. Existing analgesic formulations present weak pain relief property and long onset time even with the application of occlusions e.g. Tegaderm^^TM^^.^16^ The above limitations are a result of inefficient penetration of most analgesics especially those with high molecular weight (>500 Da) and inappropriate hydrophilicity (1<log P < 4) through the intact skin.^17^ To enhance dermal penetrations of drugs, applications of lipid-based nano-carriers are proposed. Among the various lipid-based nano-carriers, liposomes are more promising because of many merits e.g. extreme biocompatibility and biodegradability and capable of encapsulating hydrophilic and lipophilic drugs simultaneously.^18^ Invasomes are a new generation of liposomes in which terpenes and ethanol were added into their structure to provide high skin penetration.^19^ The addition of optimum amount of terpenes create deformable vesicles and is an effective strategy to increase the flexibility of the lipid bilayer of skin.^20^ Toxicity evaluation of terpenes as one of the main components of invasome indicates that natural terpenes are considered to be nontoxic.^21^ The design of an ideal invasomal formulation led to advances in the treatment of diseases such as acne and hyperpigmentation disorders and benign prostatic hyperplasia.^22-24^ For better therapeutic effects, the amount of drug that is encapsulated in nano-carrier systems must be high to be influential in the target sites. There are various methods to enhance the encapsulation efficiency (EE) i.e. the effect of variable compositions of nano-carrier systems, the effect of drug and bilayer charge interactions and diverse nano-carrier preparation methods.^25^ Variation of these parameters leads to achieving high optimal formulations in nano-carrier systems.

To develop maximum amount of bupivacaine and buprenorphine encapsulation in the nano-invasome structure, a full factorial design was used to systematically investigate the combined influence of three variables including terpene type, terpene concentration and preparation methods on the drug EE and size distribution of the nano-invasomes.

## Materials and Methods

### Materials

Buprenorphine hydrochloride was kindly donated from Faran Shimi Pharmaceutical Co., (Tuyserkan, Iran). Bupivacaine hydrochloride was purchased from MYLAN (France). Soybean Lecithin was purchased from Lipoid Co, Ltd (Ludwigshafen, Germany). Nerolidol, Menthol, KH_2_PO_4_, H_3_PO_4,_ and ethanol were obtained from Merck Chemicals (Darmstadt, Germany). Acetonitrile and methanol in HPLC grade were purchased from Duksan Co., Ltd., (Ansan, South Korea). Ultrapure water was obtained from a Milli-Q water system. (Darmstadt, Germany).

### Preparation of bupivacaine and buprenorphine-loaded invasome

Thin layer and ethanol injection methods are two common ways applied for preparation of invasome. These methods were described as follows.

### Thin layer method

First, lecithin (200 mg), buprenorphine hydrochloride (40 mg) and terpene or mixture of terpenes (variable up to 4 mg) were dissolved in 2 mL of absolute ethanol. Next, the resulting solution was transferred to a round-bottom flask and the full solvent evaporation process was carried out. Thin film formation was performed using a rotary evaporator (Teif Azma Teb, Iran) for 30 minutes. Then, the dried thin film was hydrated through stirring and vortex process using an aqueous solution (10 mL) of bupivacaine hydrochloride (40 mg) at 60°C. The resulting solution was homogenized by (SilentCrusher M, Heidolph, Germany) at 20 000 rpm. Finally, the nano-invasome was produced and stored in the refrigerator for the next experiments.

### Ethanol injection method

Briefly, lecithin (200 mg), terpene or mixture of terpenes (variable up to 4 mg) and buprenorphine hydrochloride (40 mg) were dissolved in ethanol and injected slowly into the 10 mL aqueous solution of bupivacaine hydrochloride (40 mg) under stirring condition by homogenizer at 20000 rpm.

### Experimental design

Designing novel formulations need to alter different parameters simultaneously which is a complex task and time-consuming. Therefore, using an experimental design method would be helpful in creating an ideal pharmaceutical formulation. A full factorial design is a statistical analyzing alternative to manage complex formulations in pharmaceutical designs. Random orders of different influential parameters in the invasome formulations were considered and the designed matrix with effective parameters by single replicate was carried out. The variables were as follows: terpene type (menthol or nerodilol and the mixture of them), terpene concentration (0.5, 1 and 2 w/w %) and preparation method (thin layer and ethanol injection method). Size and EE of bupivacaine and buprenorphine loaded in the invasomes were assessed as the responses. Achieving minimum particle size and obtaining high EE of two drugs are the desired plans for this step. The statistical analyses of the data were carried out by the Minitab^®^ 14 software (Minitab Inc., State College, PA).

### Bupivacaine and Buprenorphine encapsulation efficiency

First, 1 mL of the freshly prepared nano-invasomes were transferred into the Amicon^®^ ultra centrifugal filter (molecular weight cutoff = 30 kDa, Millipore, UK) and centrifuged (Universal 320 centrifuge, Hettich, Germany) for 3 minutes at 1008 g. The resulted clear solution at the bottom compartment of Amicon^®^ was injected into RP-HPLC–UV system (Knauer, Germany) to determine the concentration of unloaded drugs in the invasome structure simultaneously with the developed assay method described as follow. Analytical C_18_ column (10 µm particle diameter, 4.6 mm i.­ d. × 25 cm) (Knauer, Germany) was applied at room temperature (25°C). The absorption wave number of bupivacaine and buprenorphine was considered 210 nm. The mobile phase was composed of acetonitrile-phosphate buffer consist of 83:17 (V/V), (10 mM, pH=6.2) at the flow rate of 1 mL/min. The calibration curve was plotted in the concentration range of 5-100 µg/mL. The EE and LC were calculated as follows:

$$\mathrm{Encapsulation}\;\mathrm{efficiency}\;EE\left(\%\right)=\frac{W_{\left(Initial\;drug\right)}-W_{\left(Free\;drug\right)}}{W_{\left(Initial\;drug\right)}}\times 100$$

$$\mathrm{Loading}\;\mathrm{capacity}\;\mathrm{LC}\;\left(\text{\%}\right)\;=\frac{W_{\left(entrapped\;drug\right)}}{W_{\left(Total\;lipid\right)}}\times 100$$

#### Particle size and morphology survey

Dynamic light scattering (DLS) system (SALD 2101, Shimadzu, Japan), was applied to report the particle size distribution for prepared different invasome formulations. The data were reported as intensity-weighted average (z average) and the polydispersity index which regarded the size and distribution width of the invasome in the formulations. Size distribution was calculated through the following equation:

$$Span=\;\frac{D_{90\%}-D_{10\%}}{D_{50\%}}$$

Where D_10%_, D_90%,_ and D_50%_ refer to the percentage of the particles acquire 10%, 90% and 50% of the diameter lower or equivalent to the given value.

### X-ray diffraction and FTIR spectroscopy

Powder X-ray diffraction (XRD) was accomplished by (D5000, Siemens, Germany) with a Cu tube anode in the range of (4-40 2Ɵ). The Fourier transform infrared (FTIR) spectroscopy was carried out by (Tensor 27, Bruker, Germany) to characterize drug-loaded invasomes.

## Results and Discussion

### Preparation of bupivacaine and buprenorphine-loaded invasomes

Different bupivacaine and buprenorphine-loaded invasome formulations were prepared by full factorial experimental design and presented in Table 1. Thin layer and ethanol injection are two conventional invasome preparation methods which are slightly easy without any complexity in the preparation steps. Solvent evaporation and hydration of constructed thin layer are important steps of the thin layer method. whereas, mechanical homogenizing of aqueous media by the gradual addition of organic phase into aqueous medium are inseparable steps in the ethanol injection method. Reproducibility of the prepared formulations with favorite properties i.e. high EE and desired vesicular size needed for clinical applications are quite a challenge because of different parameters interfering in the nano-invasome properties such as preparation methods, terpene type, and terpene concentration. Scaling up, predictions and effect of the influential preparation parameters are major problems practically. Hence, identifying the related parameters and also saving advanced resources needs more information.

**Table 1:** Influential factors and obtained experimental responses for different formulations

**Test**	**Terpene type**	**Terpene Conc. (%)**	**Method**	**Size (µm)**	**Bupivacaine (EE)%**	**Bupivacaine (LE)%**	**Buprenorphine (EE)%**	**Buprenorphine (LE)%**
1	Nerolidol	2.0	Ethanol injection	0.39 ± 0.32	94.19 ± 0.52	18.8 ± 0.24	98.77 ± 2.03	19.75 ± 0.81
2	Nerolidol	2.0	Thin layer	5.22 ± 0.22	91.07 ± 1.20	18.2 ± 0.75	94.96 ± 1.64	18.9 ± 0.39
3	Menthol	2.0	Ethanol injection	0.99 ± 0.18	92.14 ± 0.45	18.4 ± 0.19	97.53 ± 1.43	19.5 ± 0.96
4	Menthol	2.0	Thin layer	4.86 ± 0.30	24.36 ± 0.89	4.8 ± 0.72	96.95 ± 0.98	19.3 ± 0.72
5	Mix	2.0	Ethanol injection	0.92 ± 0.27	79.73 ± 0.68	15.9 ± 0.3	92.40 ± 0.85	18.4 ± 0.8
6	Mix	2.0	Thin layer	5.01 ± 0.56	73.90 ± 0.67	14.7 ± 0.45	88.40 ± 0.74	17.6 ± 0.41
7	Nerolidol	1.0	Ethanol injection	1.13 ± 0.48	95.18 ± 2.65	19.03 ± 1.77	97.73 ± 0.43	19.5 ± 0.39
8	Nerolidol	1.0	Thin layer	5.73 ± 0.19	82.95 ± 0.94	16.5 ± 0.4	92.55 ± 0.56	18.5 ± 0.31
9	Menthol	1.0	Ethanol injection	1.34 ± 0.45	83.10 ± 0.82	16.6 ± 0.65	91.64 ± 0.34	18.3 ± 0.4
10	Menthol	1.0	Thin layer	5.60 ± 0.31	58.95 ± 0.78	11.7 ± 0.55	92.49 ± 2.03	18.4 ± 1.21
11	Mix	1.0	Ethanol injection	1.46 ± 0.27	78.66 ± 1.01	15.7 ± 0.89	88.92 ± 1.84	17.7 ± 0.5
12	Mix	1.0	Thin layer	4.07 ± 0.37	77.39 ± 2.04	15.4 ± 1.03	90.24 ± 0.64	18.04 ± 0.37
13	Nerolidol	0.5	Ethanol injection	0.65 ± 0.51	87.06 ± 0.69	17.4 ± 0.22	95.67 ± 0.24	19.1 ± 0.21
14	Nerolidol	0.5	Thin layer	4.56 ± 0.29	77.70 ± 0.83	15.5 ± 0.64	90.60 ± 0.15	18.1 ± 0.1
15	Menthol	0.5	Ethanol injection	0.88 ± 0.38	93.64 ± 0.93	18.7 ± 0.51	98.04 ± 0.18	19.6 ± 0.13
16	Menthol	0.5	Thin layer	5.86 ± 0.26	43.97 ± 1.57	8.7 ± 0.9	83.53 ± 0.21	16.7 ± 0.17
17	Mix	0.5	Ethanol injection	0.83 ± 0.25	88.88 ± 3.01	17.7 ± 1.89	95.83 ± 3.60	19.16 ± 2.23
18	Mix	0.5	Thin layer	5.67 ± 0.51	63.38 ± 1.45	12.6 ± 0.87	86.46 ± 0.63	17.2 ± 0.51

### FTIR Characterization

**Figure 2:**
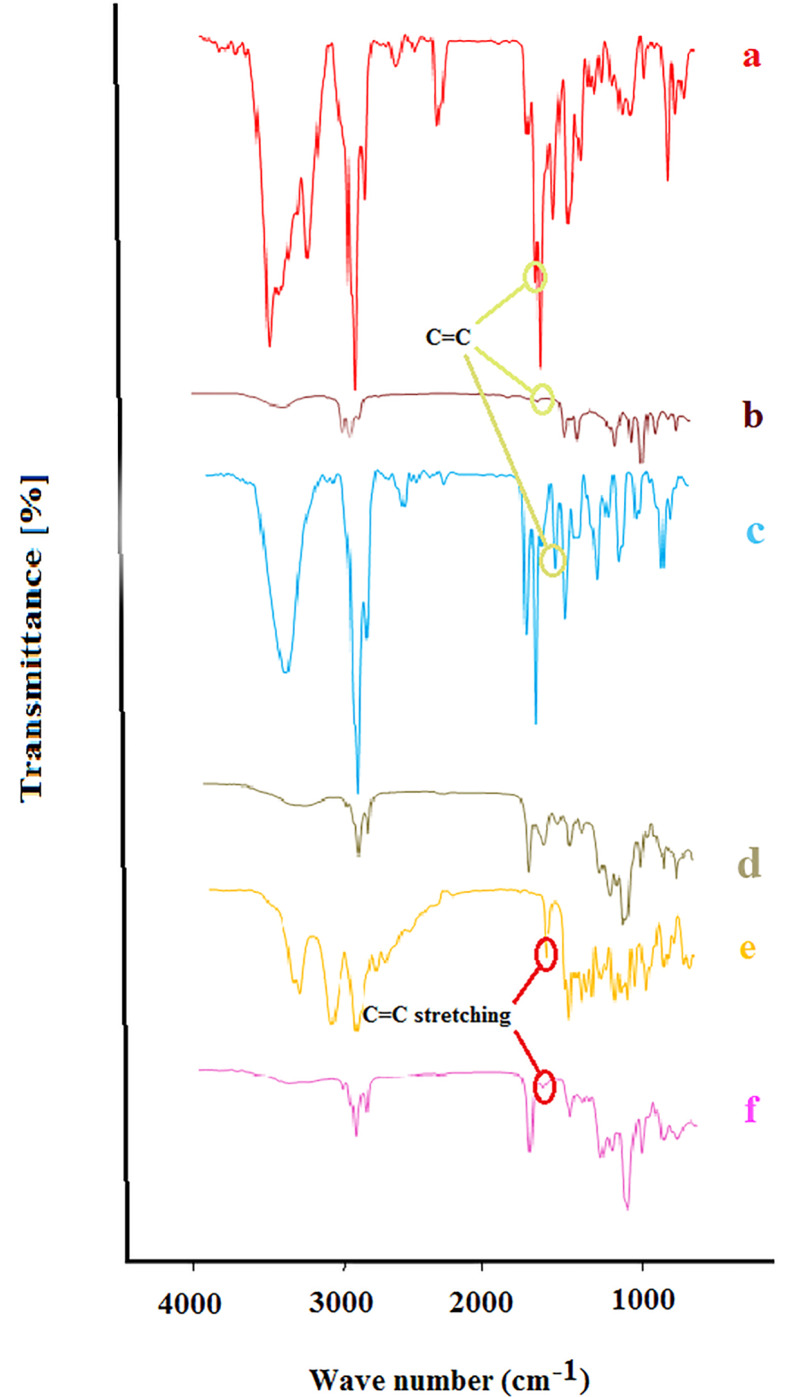
Fourier transform spectroscopy (FTIR) of (a) Bupivacaine, (b) nerolidol, (c) Bupivacaine loaded invasome, (d) blank nano-invasome, (e) Buprenorphine and (f) buprenorphine loaded invasome.

Figure 2 shows the FTIR spectra of bupivacaine (a), nerolidol (b), bupivacaine-loaded invasome (c), blank nano-invasome (d), buprenorphine (e) and buprenorphine-loaded invasome (f). Presence of a shift in the bond peak of C=C in 1688 cm-^1^ of Figure 2a to 1642 cm-^1^ in Figure 2b demonstrates π-π electron system interaction of nerolidol in the invasome structure with a double bond of C=C groups on the bupivacaine that indicates the well loading of bupivacaine into the nano-invasome chemical structure. The study of the FTIR spectrum of the buprenorphine in Figure 2e shows a peak bond at 1640 cm-^1^ which is related to the stretching vibrations of C=C. Presence of a considerable decrease in the intensity of the C=C peak bond in 1645 cm-^1^ related to buprenorphine loaded invasome in Figure 2f confirms the well interaction of buprenorphine with the phospholipids of the nano-invasome chemical structure.

### X-ray diffraction

**Figure 3:**
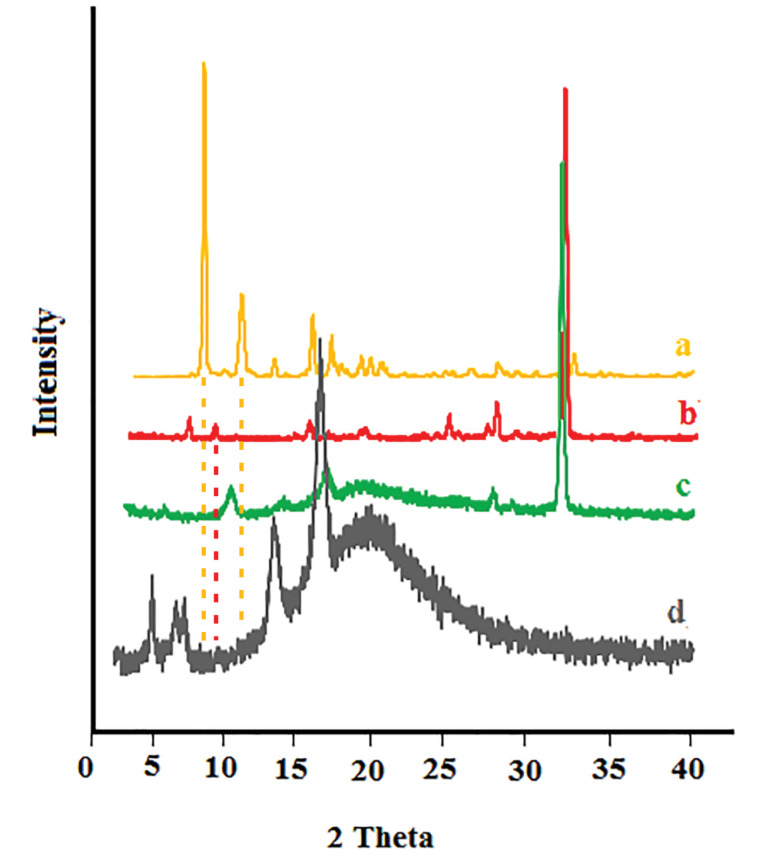
X-ray diffraction pattern of (a) buprenorphine, (b) bupivacaine, (c) drug-loaded nano-invasome and (d) blank nano-invasome.

The presence of sharp peaks in the diffraction X-ray pattern of buprenorphine and bupivacaine confirmed the crystalline nature of the drugs (Figure 3a, 3b), respectively. The absence and a significant reduction in the intensity of the main peaks of buprenorphine and bupivacaine in the invasome structure confirmed the formation of amorphous pattern in the drug-loaded invasome structures (Figure 3c). The X-ray diffraction pattern of blank nano-invasome is shown in Figure 3d. The comparison of the diffraction pattern of buprenorphine, bupivacaine, blank nano-invasome and drug-loaded invasome structures verified molecularly and uniformly dispersion of the drugs in the nano-invasome structures. The amorphous distribution of the drug in the carrier structure provides more capacity for a carrier for drug loading. The description above may interpret high observed LC value for both drugs. Furthermore, drugs in the amorphous structure show faster dissolution than crystalline ones which enhance their therapeutic performance in the site of action.^26^

### Encapsulation efficiency

**Figure 4:**
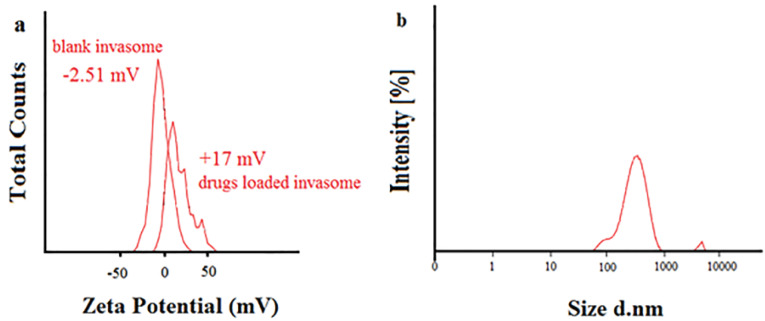
Zeta potential of (a) blank nano-invasome and drugs-loaded nano-invasome, (b) Size of the optimum formulation.

**Figure 5:**
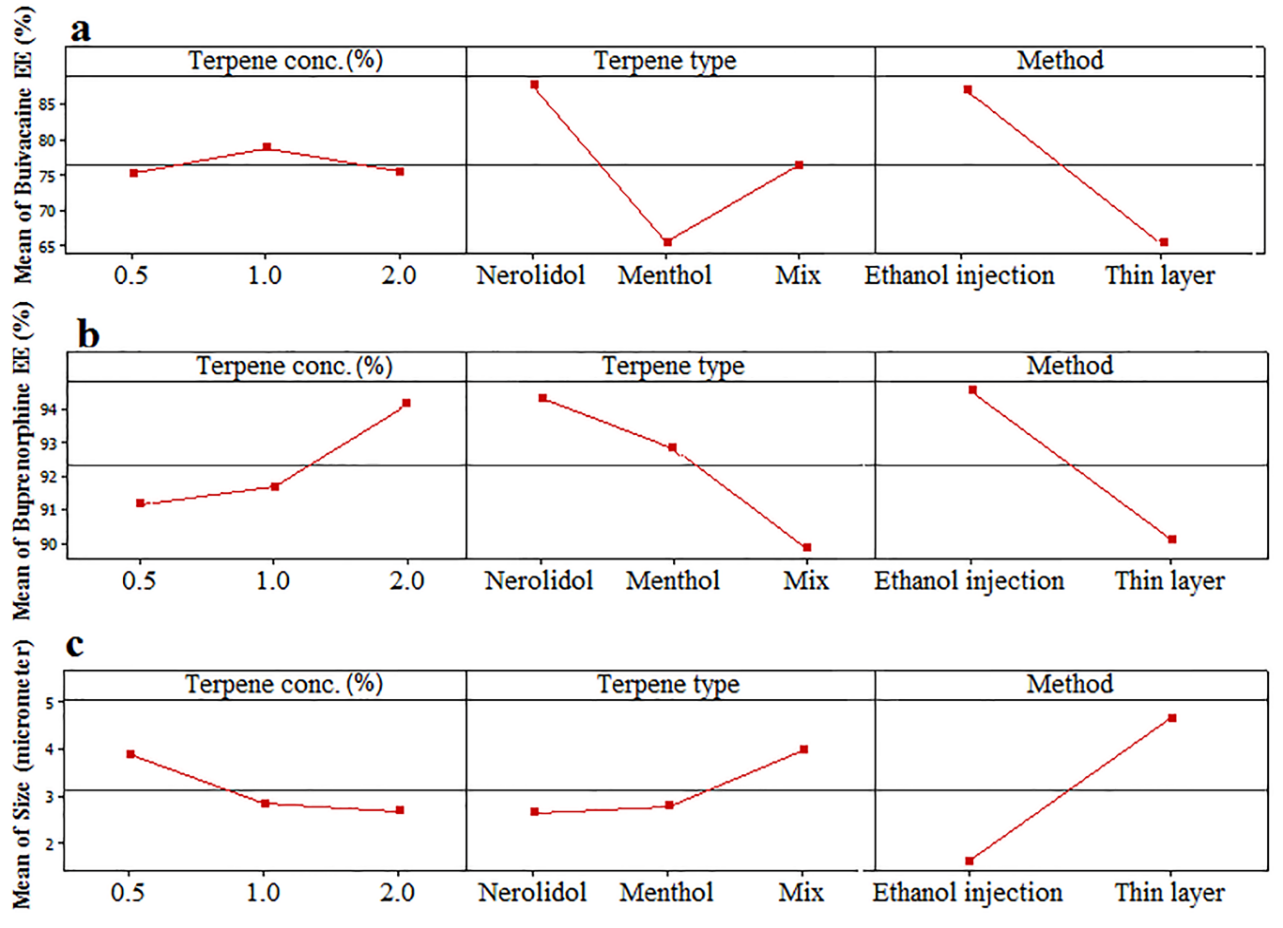
Effect of variables including terpene type (Nerolidol, Menthol, Mix), terpene concentration (0.5, 1, 2%) and method of preparation on the EE of (a) bupivacaine, (b) buprenorphine and (c) size of vesicles.

**Figure 6:**
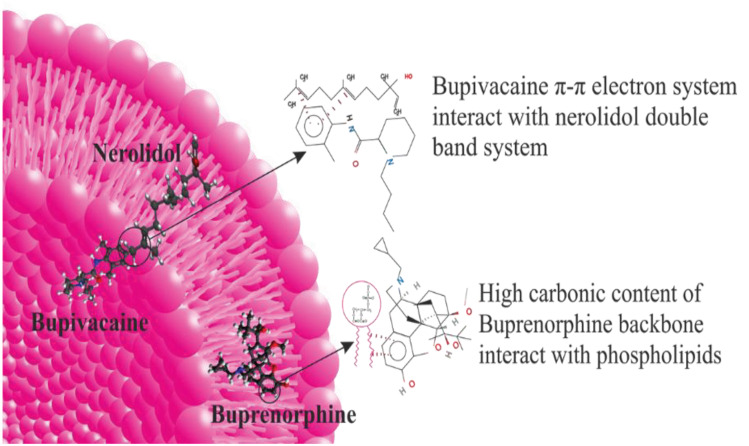
Schematic of chemical interactions between drugs and nano-invasome structure.

**Figure 7:**
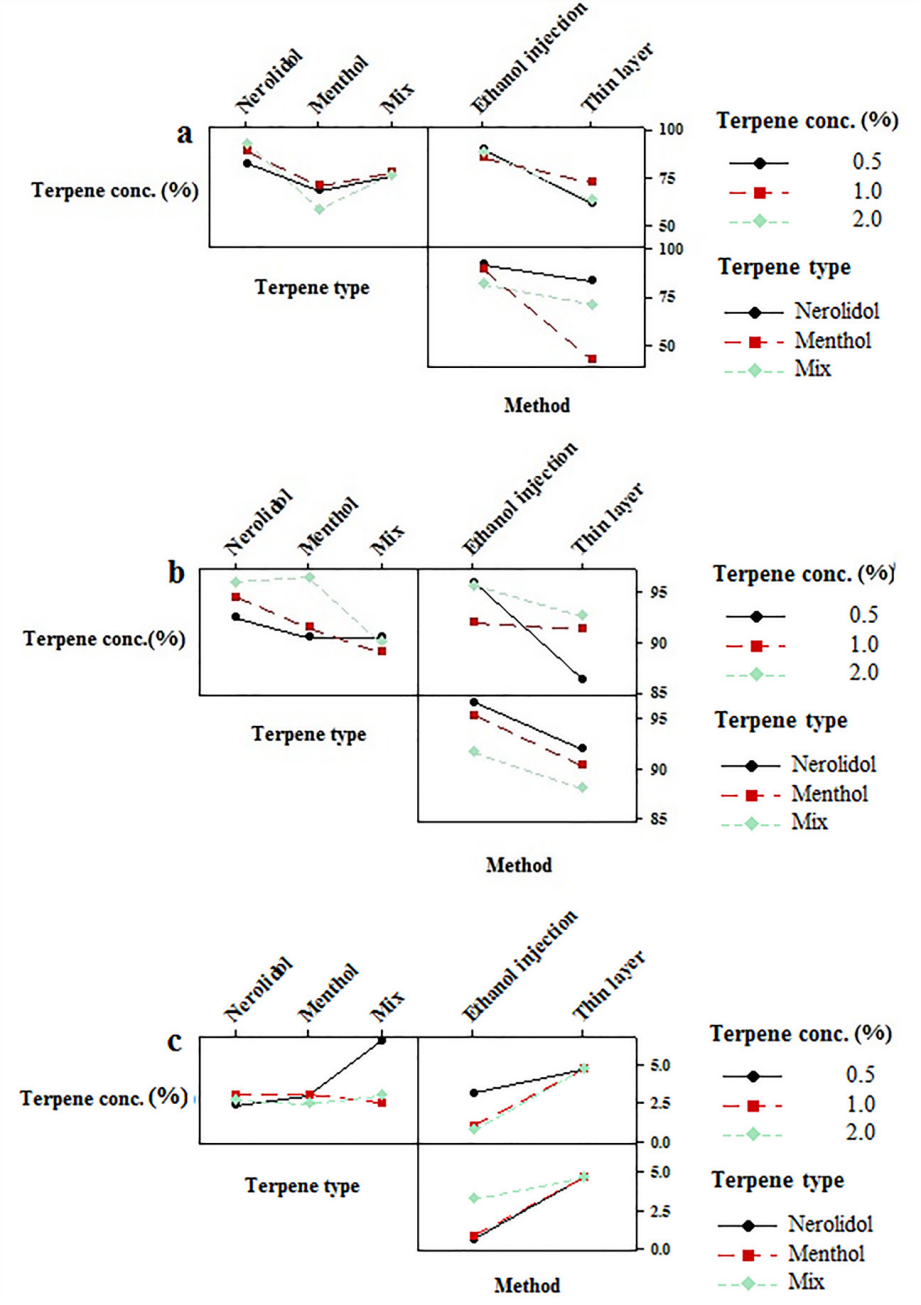
Interaction plots for EE of (a) bupivacaine, (b) buprenorphine and (c) size of vesicles.

EE percentage was evaluated by high-performance liquid chromatography coupled with ultraviolet detector (HPLC-UV) technique and the results are shown in Table 1. High values of EE and LC were reported as 98.77, 95.18 % and 19.75, 19.03 %, respectively for both buprenorphine and bupivacaine-loaded invasome.^27^ The obtained value of EE for hydrophilic buprenorphine and bupivacaine-loaded invasome system is comparable with the amount of EE for some lipophilic drugs-loaded invasome systems. This might be as a result of reversible ionic interactions between positively charged buprenorphine and bupivacaine hydrochlorides and negatively charged phospholipid part of invasome structure. To designate the reason, zeta potential measurements were carried out for blank and drug-loaded invasome structures (Figure 4a). As the results show, the blank invasome presents -2.51 mV zeta potential value whereas, the zeta potential value for drugs-loaded invasome was measured as +17 mV. The clear difference between the zeta potential values of blank and drug-loaded invasome strongly confirms the electrostatic interactions mentioned above. A comprehensive study of influential factors in the invasome preparation indicates that EE of bupivacaine is affected by the preparation method and type of terpenes i.e. nerolidol (Figure 5a). According to the chemical structure of bupivacaine, there is no effective interaction between menthol and bupivacaine. While chemical structure of Bupivacaine consists of π-π electron system which well interacts with the nerolidol double band system (Figure 6a). This process results in achieving higher EE in both thin layer and ethanol injection methods. According to the results, buprenorphine EE was not influenced by terpene type, terpene concentration and preparation methods (Figure 5b). High carbonic content of the buprenorphine backbone encourages well interaction of buprenorphine and phospholipids tail which develops the EE spontaneously (Figure 6b). Therefore, altering different preparation factors do not play an important role in the EE of buprenorphine. F2 and F4 formulations containing nerolidol 2% and menthol 2% which were prepared with a thin layer method resulted in EE of 91.07 and 24.36 % for bupivacaine, respectively. F1 and F11 formulations containing nerolidol 2% and 1% mixture of terpenes, present EE of 94.19, 78.66 % for bupivacaine in ethanol injection method, respectively. The reported data above are for the maximum and minimum value of EE for bupivacaine in thin layer and ethanol injection methods. Interactions between terpene concentration, terpene type and method of preparation on the EE of bupivacaine and buprenorphine are shown in Figures 7a and 7b, respectively. In all applied terpene concentrations using the ethanol injection method, there is an overall improvement in the EE% (Figure 7a, 7b). As it is clear in the Figure 7b, the incorporation of terpenes concentration effect led to the dominant effect of preparation methods on the EE. The ethanol injection method showed better EE for bupivacaine than thin layer method probably due to the presence of ethanol in the invasome structure providing higher flexibility than conventional liposomes, which possibly enhances EE of hydrophilic drugs.^28^ Therefore, among two various preparation methods, ethanol injection was presented superior EE than the thin layer method (Table 2). The obtained result is in good accordance with the literature.^25^

**Table 2:** Influential factors affecting optimum formulation conditions

**Influential factors**	**Hight encapsulation**		**Optimum size**
	**Bupivacaine**	**Buprenorphine**	
Preparation Method	Ethanol Injection	Ethanol Injection	Thin Layer
Terpene Type	Nerolidol	Nerolidol	Menthol
Terpene Concentration	2%	2%	0.5

### Size characteristics of bupivacaine and buprenorphine loaded invasome

Particle size is an important character of carries which may promote dermal penetration capability that leads to a high concentration of drugs in the target site.^29,30^ Therefore, the evaluation of influential formulation factors is crucial for designing invasomes with optimum sizes (Figure 4b). The mean sizes of designed invasomes are in the range of 0.39-5.86 µm that are presented in Table 1 which illustrated the significant effect of variables on the size. The preparation method showed the most influencing effect on the size than terpene type and concentration. Ethanol injection method provided lower particle size probably due to the presence of ethanol in the structure which increases the flexibility of vesicular structure and better affecting from shear forces for size reduction. This fact has been reported for ethosomes in comparison to the conventional liposomes.^28^ The presence of terpene in high concentrations caused more decrease in the size of invasomes (Figure 5c). The obtained data are in contrast with the other reports.^28^ This phenomenon might be related to the variety of the terpenes type, applied in the present nano-invasome formulation. Table 2 presents the maximum and minimum size of invasomes obtained under the influence of factors to design an ideal formulation.

## Conclusion

Two groups of opioid and non-opioid analgesic drugs such as buprenorphine and bupivacaine hydrochloride were used for simultaneous loading into a novel nano-invasome system for dermal delivery. Invasomes are promising vesicular systems which enhance the dermal penetration in comparison to liposomes due to the presence of terpenes and ethanol in their structures. Characterization of designed formulations were carried out by different techniques such as FTIR, XRD, zeta potential and DLS. High EE values of buprenorphine and bupivacaine hydrochloride were achieved in the invasome structure because of ionic interactions and non-covalent chemical bonding between the drugs and nano-invasome compositions. Among two simultaneous loaded drugs, bupivacaine was more affected by the preparation method and terpene type. Nerolidol and ethanol injection method showed higher EE and lower vesicular size for both drugs.

## Ethical Issues

Not applicable.

## Conflict of Interest

All Authors declare no conflict of interest.

## Acknowledgments

The authors would like to acknowledge from Drug Applied Research Center and Faculty of Advanced Medical Science of Tabriz University of Medical Science for supporting this project. (Grant NO. 59576).
